# Highly sensitive H_2_S sensors based on Cu_2_O/Co_3_O_4_ nano/microstructure heteroarrays at and below room temperature

**DOI:** 10.1038/srep43887

**Published:** 2017-03-02

**Authors:** Guangliang Cui, Pinhua Zhang, Li Chen, Xiaoli Wang, Jianfu Li, Changmin shi, Dongchao Wang

**Affiliations:** 1 Institute of Condensed Matter Physics, Linyi University, Linyi, Shandong 276000, China; 2 School of science, Linyi University, Linyi, Shandong 276000, China

## Abstract

Gas sensors with high sensitivity at and below room temperature, especially below freezing temperature, have been expected for practical application. The lower working temperature of gas sensor is better for the manufacturability, security and environmental protection. Herein, we propose a H_2_S gas sensor with high sensitivity at and below room temperature, even as low as −30 °C, based on Cu_2_O/Co_3_O_4_ nano/microstructure heteroarrays prepared by 2D electrodeposition technique. This heteroarray was designed to be a multi-barrier system, and which was confirmed by transmission electron microscopy, scanning electron microscopy, X-ray photoelectron spectroscopy and scanning probe microscopy. The sensor demonstrates excellent sensitivity, sub-ppm lever detection, fast response, and high activity at low temperature. The enhanced sensing property of sensor was also discussed with the Cu_2_O/Co_3_O_4_ p-p heterojunction barrier modulation and Cu_2_S conductance channel. We realize the detection of the noxious H_2_S gas at ultra-low temperature in a more security and environmental protection way.

Conventional gas sensing based on the change of carrier concentration caused by adsorption/desorption of adsorbed oxygen on the surface determines the disability of sensor at extreme conditions, e.g., ultra-low temperature and low gas concentration. First, the temperature dependence of adsorption/desorption process leading to a high working temperature of sensors, which is harmful for the manufacturability, security and environmental protection, high-temperature operation is also undesirable in many situations, particularly in an explosive environment where high temperatures could trigger an explosion^1^,^2^,^3^. Second and more important, the test signal (i.e. the change of conductivity caused by variation of carriers’ concentration) is very limited if the adsorption and desorption process is weak at extreme conditions. And unfortunately, the linear relationship between the variation of carriers’ concentration and the magnitude of adsorption/desorption process is unbreakable. However, the significant enhancement of conductivity caused by a weak variation of carriers’ concentration can be achieved by interface effect of heterostructure^4^,^5^.

There have been considerable efforts to improve sensors’ performance under extreme conditions, such as using nanostructured materials with ultra-high surface-to-volume ratios, appropriate element doping, surface decoration with noble metals and construction of heterostructure^6^,^7^,^8^,^9^. Among them, heterostructure materials have been confirmed to be ideal materials for low temperature gas detection. The conductivity of heterostructure materials mainly depends on the heterojunction barrier, which is sensitive to the carrier concentration. Hence, a significant enhancement of conductivity can be obtained even in case of a weak change of carrier concentration. Therefore, heterostructure materials have the ability to perceive slight change of surface absorption under extreme conditions^10^. Nevertheless, production of sensors operating at lower temperature (especially below freezing temperature) with high sensitivity, fast response, and low power consumption remains a challenging task.

Along with the advance of production security and living environmental awareness, the detection of flammable and toxic gases becomes one of the noticeable research topics^11^. Hydrogen sulfide (H_2_S) is one of the most toxic gases and possess a health risk at high concentrations as apart from its unpleasant smell even at relatively low concentration^12^. In addition, H_2_S is corrosive, flammable, and explosive, and the lower explosive limit of H_2_S for flammability is approximately 4%^13^. So the high working temperature of sensor is a major threat for H_2_S detection. To date, several types of sensor, such as porous CuO nanosheets, CuO-ZnO nanorods, SnO_2_ quantum wire/reduced graphene oxide nanocomposites and Ag_2_O/SnO_2_ ordered mesoporous, have been utilized for H_2_S gas detection at low temperature^14^,^15^,^16^,^17^. In addition, heterostructure materials show many advantages due to their advantages in conductivity modulation at low temperature^4^,^18^. Among the investigated metal oxide sensing materials, the p-type Cu_x_O semiconductor has been extensively researched because of its reversible reaction with H_2_S^19^. And the reaction product metallic Cu_x_S give rise to a great change of conductivity, especially for heterostructures based on Cu_x_O. Many Cu_x_O based heterostructure materials have been proposed and achieved some progress in H_2_S gas detection at low temperature^20^,^21^. However, fabrication of gas sensors with lower working temperature is still highly expected.

Heterostructure materials with strictly periodic arrangement in millimeter range are ideal candidates for research and nano-microdevice applications. Hence, great attention has been paid to design of new heterojunction architectures to enhance the sensing capability of the sensor^22^. To improve the performance, we propose a Cu_2_O/Co_3_O_4_ nano/microstructure heteroarrays with strictly periodic structure in millimeter range composed of nanojoint and nanowire. Different from previous reported heterojunction materials based on Cu_2_O/CuO and Co_3_O_4_, this heteroarray was designed to be an ordered multi-barrier system with its application based on interface field modulation. The combined effect of both modulation of interface field and formation of metallic Cu_2_S can induce electrical properties distinct from their individual impact, thereby realizing synergistic performance (“1 + 1 > 2” or “more than the sum of its parts”).

## Results

**Figure 1:**
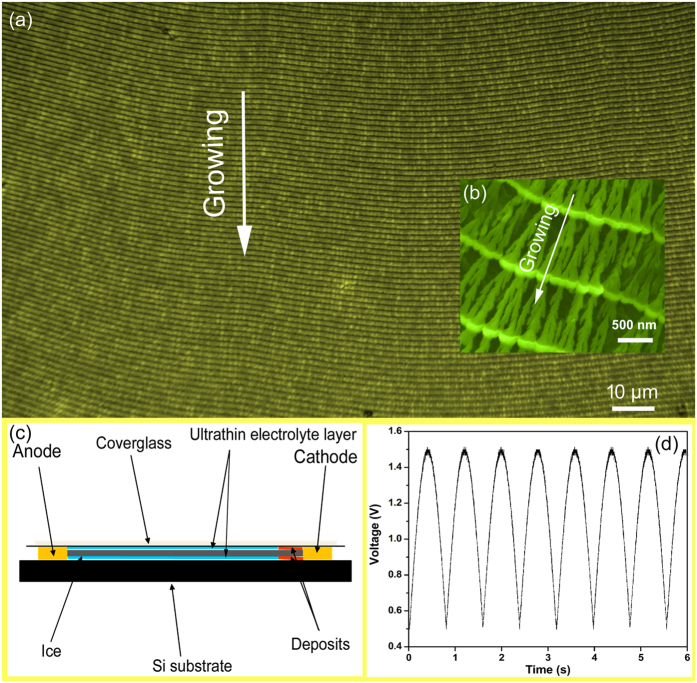
Morphology and experimental details of Cu_2_O/Co_3_O_4_ nano/microstructure heteroarrays. (**a**) Image of heteroarrays captured by an optical microscope at 500× magnification, the pattern exhibits uniform cycle length and good long-range order. (**b**) SEM image at high magnification showing the periodic structure of heteroarrays. (**c**) Schematic diagrams showing the construction of the ultra-thin electrolyte layer. (**d**) Semi-sine waves applied across the electrodes in the electrochemical deposition process.

We have made a Cu_2_O/Co_3_O_4_ nano/microstructure heteroarrays using 2D electrodeposition by applying a semi-sine wave potential in quasi-2D ultra-thin liquid layer. Figure 1a captured by an optical microscope at 500× magnification, showing a top view of the heteroarrays over a large area, the bamboo-like pattern exhibits uniform cycle length and good long-range order. The substrate was nearly 60% covered by the sample, and the area of a single domain can reach a few square millimeters, it means the biggest heteroarrays with strictly periodic arrangement have dimensions in millimeters. The detailed organization of the heteroarrays was investigated by scanning electron microscopy (SEM), as shown in Fig. 1b. As shown in SEM image, each cycle is composed of a nanojoint and a nanowire. The good long-range order in millimeters of resulting patterns extend their utility in some applications. These figures provide additional evidence that 2D electrodeposition is an ideal method to synthesize heteroarrays with ordered nano/microstructure in millimeter range without template. The construction of quasi-2D ultra-thin electrolyte layer was shown in Fig. 1c, and the thickness of the ultra-thin layer is about 300 nm. The waveform of the applied voltage is found to be semi-sine with a frequency of 0.8 Hz and amplitude varied between 0.5 and 1.5 V, as shown in Fig. 1d. The waveform supplied by an arbitrary waveform generator.

**Figure 2:**
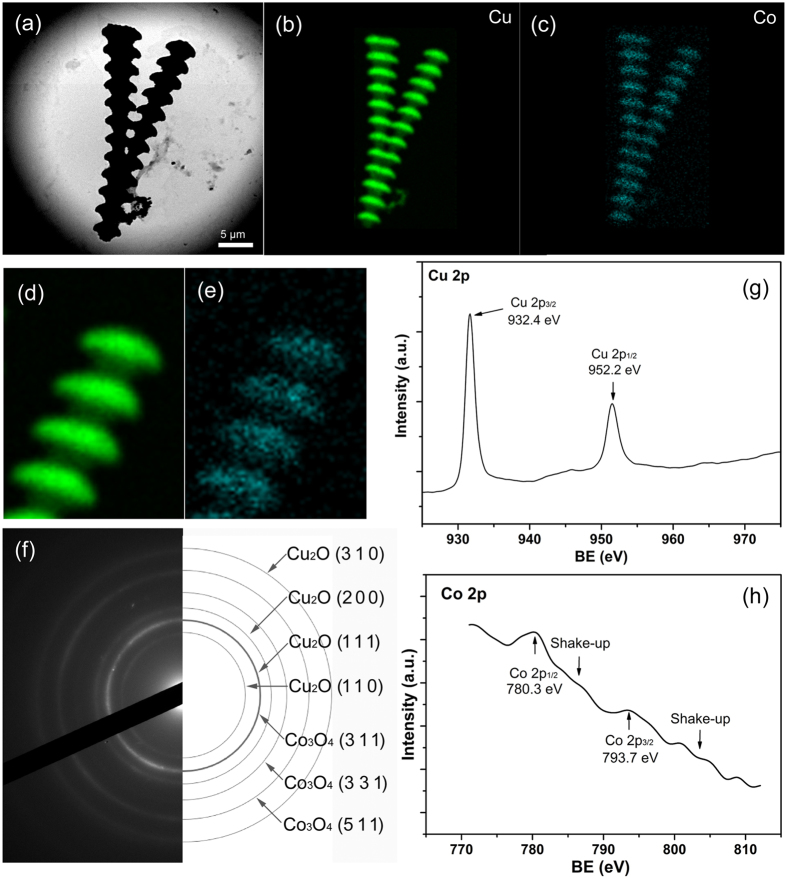
Structure and component of Cu_2_O/Co_3_O_4_ nano/microstructure heteroarrays. (**a**) TEM image of the heteroarrays. (**b**) and (**c**) are the distribution of Cu and Co corresponding to (**a**). (**d**) and (**e**) are magnification images of Cu and Co distribution, showing the continuous distribution of Cu and heterogeneous distribution of Co. (**f**) SAED pattern of heteroarrays, the diffraction rings are corresponding to Cu_2_O and Co_3_O_4_. (**g**) and (**h**) are curve-fitting results of the Cu 2p and Co 2p XPS spectra.

Structure and component of Cu_2_O/Co_3_O_4_ nano/microstructure heteroarrays were confirmed by Transmission electron microscope (TEM) and X-ray photoelectron spectroscopy (XPS). The TEM image further reveals the bamboo-like morphology of heteroarrays (Fig. 2a). Element-distribution mapping in Fig. 2b and c corresponding to Fig. 2a show the distribution of Cu and Co, and local enlarged images of Cu and Co distribution are shown in Fig. 2d and e respectively. We can see that the Cu is distributed throughout the whole sample, but the Co is heterogeneous. We can conclude that the Co_3_O_4_ is located mainly at the nanojoint and discrete at the nanowire. The selected area electron diffraction (SAED) pattern of heteroarrays (Fig. 2f) demonstrates a typical ring structure characteristic for polycrystalline materials and agrees well with the structure of Cu_2_O and Co_3_O_4_. The atomic planes (110), (111), (200), (310) of Cu_2_O and (311), (331), (511) of Co_3_O_4_ can be fully indexed. The high resolution transmission electron microscopy (HRTEM) image of Cu_2_O/Co_3_O_4_ nano/microstructure heteroarrays was characterized and supplied in Fig. S1. The spacings of the fringes were measured to be 0.25 nm for Co_3_O_4_ and 0.3 nm for Cu_2_O, corresponding to the (311) plane of Co_3_O_4_ and the (110) plane of Cu_2_O. The results well agree with the SAED. Furthermore, Co_3_O_4_ and Cu_2_O are tightly contacted with each other, and the boundary is distinct. The elemental composition and chemical states of the heteroarrays were further confirmed using XPS. The binding-energy values at 932.4 eV and 952.2 eV represent Cu 2p in the nanocomposites, as shown in Fig. 2g. This result indicates that the Cu exists in a Cu^+^ oxidation state^23^. The XPS spectra of Co peaks is presented in Fig. 2h. Their consistent binding energies (780.3 eV, 793.7 eV and shake-up peaks) represent the integrated peaks of Co_3_O_4_^24^.

**Figure 3:**
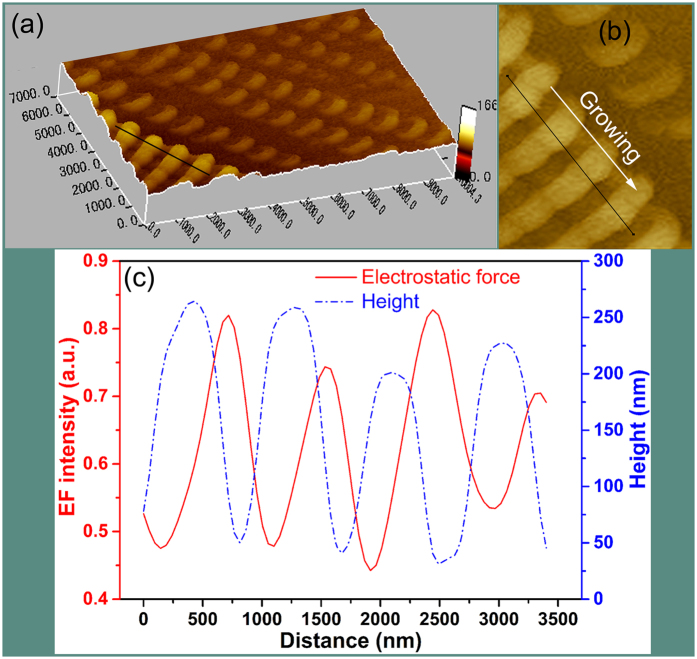
The interface electrostatic field distribution of Cu_2_O/Co_3_O_4_ nano/microstructure heteroarrays. (**a**) 3D stereogram of Cu_2_O/Co_3_O_4_ nano/microstructure heteroarrays. (**b**) The top view of heteroarrays present the test zone of electrostatic force in (**a**), and the line-profile analysis path is marked by black line simultaneously in (**a**) and (**b**). (**c**) The distribution of electrostatic field along with the variation of height of heteroarrays.

Figure 3 shows 3D stereogram image and electrostatic field distribution of Cu_2_O/Co_3_O_4_ nano/microstructure heteroarrays characterized by Scanning Probe Microscope (SPM). SPM with electrostatic field microscope (EFM) function allows the measurement of electrostatic field distribution^25^. The line-profile analysis path was marked by black line simultaneously in Fig. 3a (3D stereogram image of heteroarrays) and Fig. 3b (top view of the corresponding test zone in Fig. 3a). The variation in surface elevation is very regular along the growth direction, as shown in Fig. 3a. The height of nanojoint and nanowire are about 250 nm and 50 nm (Fig. 3c). Our line-profile analysis path of electrostatic force was along the growing direction. The line-profile in Fig. 3c reveals that the variation of electrostatic force has the same oscillation period as the surface elevation with a lag. In a typical cycle length, we can get the maximum value of the surface elevation first followed by the maximum of electrostatic field along the heteroarrays growing direction.

**Figure 4:**
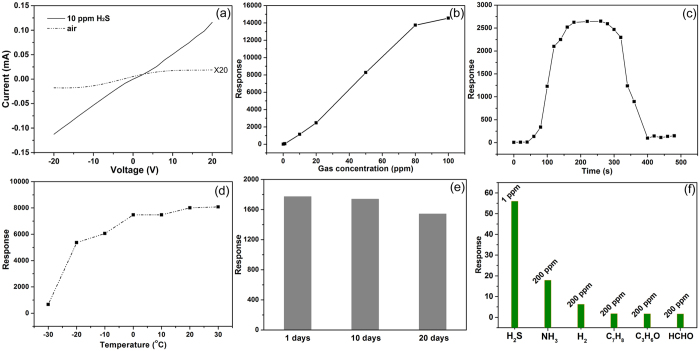
The performance of typical sensor based on the Cu_2_O/Co_3_O_4_ nano/microstructure heteroarrays. (**a**) I-V curves of the sensor in air and 10 ppm H_2_S atmosphere (the current in air was magnified 20 times). (**b**) The H_2_S concentration dependence of the sensor. (**c**) Dynamic response of the sensor to 20 ppm H_2_S in air. (**d**) The temperature dependence of the sensor to 50 ppm H_2_S in air. All the data in (**a**), (**b**) and (**c**) were recorded at 25 °C. (**e**) The response of sensor to 20 ppm H_2_S versus the storing time for 1, 10 and 20 days at 15 °C. (**f**) The response of sensor to 200 ppm NH_3_, H_2_, C_7_H_8_, C_3_H_6_O, HCHO and 1 ppm H_2_S in air at working temperature of 15 °C.

The special structure of the Cu_2_O/Co_3_O_4_ nano/microstructure heteroarrays determines its excellent H_2_S sensing performance at room temperature. The *I-V* curves of a typical sensor in air exhibit obvious nonlinear characteristics at room temperature, indicating the presence of heterointerface barrier (Fig. 4a). Notably, the current in air is in the range of 10^−7^ A, which is low and enhances the sensitivity of the sensor^26^. The conductivity increased obviously when the sensor was exposed to 10 ppm H_2_S in air at 25 °C, while the plot of current (I) as a function of bias voltage (V) became a linear relation. It can be observed in the Fig. 4b that the sensor has a wide detection range for H_2_S from 0.1 to 80 ppm at room temperature. The response increases linearly with increasing H_2_S concentration between 0.1 and 80 ppm. Above 80 ppm, the response has no significant change, indicating that the response becomes saturated. The sensitivity of the sensor to H_2_S at 25 °C is about 180. Notably, the sensor has reasonable response (Response = 11.2) to 0.1 ppm H_2_S in air at 25 °C, which means the H_2_S detection limit of the sensor can reach as low as sub-ppm at room temperature. Figure 4c shows the dynamic response of the sensor to 20 ppm H_2_S in air at 25 °C. During exposure to H_2_S gas, the response increased and then reached saturation in about 200 s. Similarly, the response reduced as soon as the gas was turned off, and the sensor restored to its original state in 230 s. The response between −30 and 30 °C to 50 ppm H_2_S is shown in Fig. 4d. It demonstrates that the sensor has high response to 50 ppm H_2_S below room temperature, even as low as −30 °C (response = 663.2). The sensor response increased linearly with the increasing working temperature from −20 to 30 °C.

The stability is highly crucial in widening the application fields of the nanostructure materials, it is also one of the most important characteristics for sensors. The response of sensor versus the storing time is shown in Fig. 4e. After the first measurement, the sensor was stored in dry air for subsequent sensing stability tests. A series of tests were carried out at the times of 1, 10 and 20 days after the device fabrication, with a 20 ppm H_2_S at a working temperature of 15 °C. It was found that very small variations were detected in the responses of the sensor, showing that the sensor exhibited good long-term stability after the initial storage duration. The Cu_2_O and Co_3_O_4_ show excellent chemical stability in atmosphere, for which the sensors have good stability. We explored the selective detection of NH_3_, H_2_, toluene (C_7_H_8_), acetone (C_3_H_6_O) and methanol (HCHO) with the sensor, and the sensitivities are illustrated in Fig. 4f. At the exposed concentration of 200 ppm at 15 °C, the response is 18%, corresponding to NH_3_, which is 2 times lower when compared to H_2_S at 1 ppm. As for H_2_, C_7_H_8_, C_3_H_6_O and HCHO, there is no significant response signal when the exposed concentrations were 200 ppm, suggesting that our sensor is sensitive to H_2_S compared to the above gases.

## Discussion

**Figure 5:**
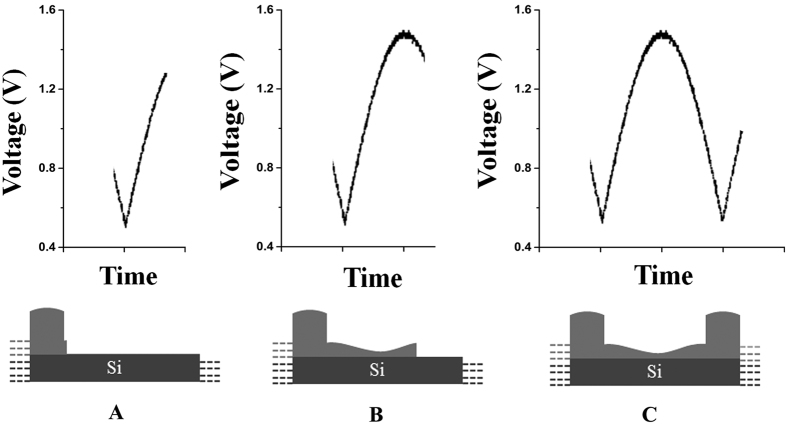
Schematic diagrams showing the periodic deposition process. The growth process at the lower and upper part of the semi-sine wave potential are corresponding to the deposition of nanojoint and nanowire respectively.

The thickness of the ultra-thin layer is about 300 nm, in which the convection and diffusion of ions are limited. That means the electromigration of ions from anode to cathode is the main activity reserved in the ultra-thin layer. Therefore, the ions can be deposited *in situ* and assembled to be ordered nanostructures^27^. The growth velocity is closely related to the concentration of electrolyte and the applied potential. The periodical pattern is caused by the variation of ion concentration near the growth interface lagging behind the variation of electrode potential^28^. In this deposition process, the Cu^2+^ and Co^2+^ ions were driven to the growth interface persistently by the electric field, where they were co-deposited. As shown in Fig. 5, when the potential is low, the ions can migrate to the growth interface in time as the deposition process is relatively slow. Therefore, the deposit are easy to accumulate, which means the nanojoint of the heteroarrays formed at this time. In contrast, the deposition process is relatively fast when the potential is high. However, the electromigration velocity of ions increased faintly, which means the supply of ions could not meet the consumption at the growth interface. Hence, the deposit are not easy to accumulate, leading to the formation of nanowires.

**Figure 6:**
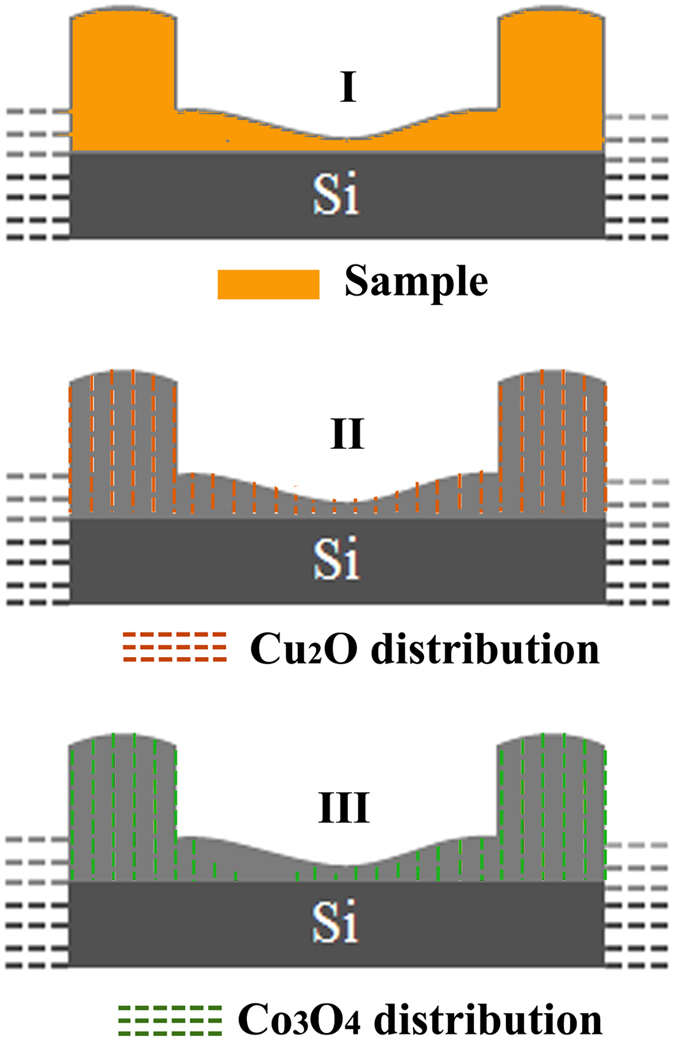
The schematic diagram of Cu_2_O and Co_3_O_4_ distribution across the longitudinal section. The Cu_2_O is distributed throughout the whole sample, but the Co_3_O_4_ is heterogeneous.

Both lower and upper part of applied semi-sine potential can realizes the co-deposit of Cu_2_O and Co_3_O_4_. However, their deposition behaviour are not identical due to the different concentration of Cu^2+^ (60 mM) and Co^2+^ (30 mM) ions. At the lower part of the potential, the Cu^2+^ and Co^2+^ ions could migrate to the growth interface in time, and they were co-deposited to be a composite of Cu_2_O and Co_3_O_4_. Meanwhile, the growth at the upper part of the potential is relatively fast, the deposit stretch forward rapidly. But only Cu^2+^ could migrate to the growth interface to a certain extent for the relatively large concentration. That means the deposition of Co_3_O_4_ is interrupted because of the relatively low concentration, as shown in Fig. 6. Hence, pure Cu_2_O nanostructure must appeared as a small section of nanowire. And the structure information shown in Fig. 2 confirm this point. In conclusion, we may declare that the Cu_2_O/Co_3_O_4_ nano/microstructure heteroarrays are composed by alternately distributed Cu_2_O and composite of Cu_2_O and Co_3_O_4_. When the charge redistribution in the Cu_2_O-Cu_2_O/Co_3_O_4_ composite heterointerface reaches equilibrium, a built-in electric field is induced^29^. However, the heterointerfaces on both sides of the pure Cu_2_O nanostructure are different. Along with the growth direction, the composite-Cu_2_O heterointerface formed first at the voltage rise process. During this period, the supply of Co^2+^ was least because of the lagging electromigration velocity. Hence, the heterointerface is relatively clear. Instead, the Cu_2_O-composite heterointerface formed subsequently at the voltage decrease process, while the supply of Co^2+^ increased gradually. Hence, the Co_3_O_4_ content gradually increase in the deposit leading to an unsharp heterointerface. Thus, the strongest electrostatic field distributed in the composite-Cu_2_O heterointerface, rather than the middle position of nanowire. The electrostatic force distribution along with the variation of height shown in Fig. 3c is the direct evidence.

**Figure 7:**
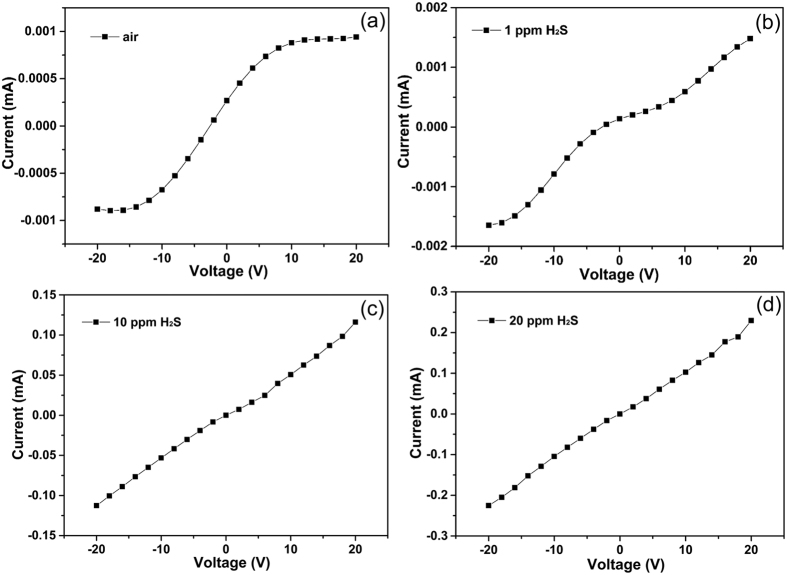
The dependence of the I-V curves’ shape on the H_2_S concentration. (**a**) I-V curve of sensor in air. Correspondingly, the I-V curves of sensor in 1 ppm, 10 ppm and 20 ppm are presented in (**b**), (**c**) and (**d**). The shape of I-V curves changed significantly when the sensor exposed in different levels of H_2_S atmosphere. All the data were recorded at 25 °C.

**Figure 8:**
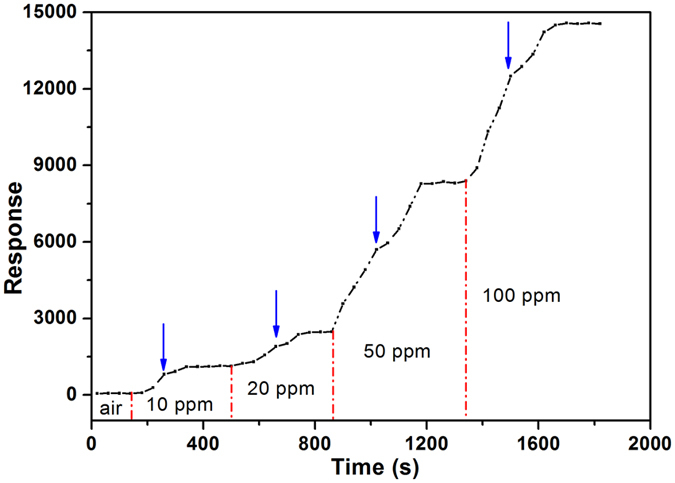
Gas responses as a function of H_2_S concentration of sensor at 25 °C. The continuous gas response at each step of H_2_S gas concentration. The data exhibit an inflection point at each growth process of response, as marked by blue arrows.

The I-V curve of the typical sensor in air exhibits obvious nonlinear characteristics at room temperature, as shown in Fig. 7a, indicating the presence of heterointerface. The investigation shown that the conductivity of the sensor increased gradually as the H_2_S concentration increases from 1 to 20 ppm. When the sensor exposed to 1 ppm H_2_S in air (Fig. 7b), the conductivity increased by about 60%, and the I-V curve remained the nonlinear characteristics. Linear relationship between I and V emerged when it was exposed to 10 ppm H_2_S (Fig. 7c), while the conductivity continued to increase as high as two orders of magnitude. And in 20 ppm H_2_S atmosphere (Fig. 7d), a standard linear relationship of I and V formed, and the conductivity is more than double that in 10 ppm H_2_S atmosphere. The change of I-V relationship (from a nonlinear to a linear character) means a variation of carriers’ transport. The details of dynamic responses of the sensor to H_2_S confirm this point. Figure 8 shows a continuous test without recoveries to 10–100 ppm H_2_S concentration range at 25 °C. Upon exposure to different concentrations of H_2_S, the response increased fast, and then reached saturation rapidly. The data exhibit that during the response growth process a two-stage response can be observed as the frontal curve of the response peak exhibits two distinguished slopes before reaching the plateau region. The inflection point at each growth process are marked by blue arrows.

The H_2_S sensors based on the Cu_2_O/Co_3_O_4_ nano/microstructure heteroarrays display excellent response at and below room temperature, even at −30 °C. We believe that both desorption of oxygen and generation of metallic Cu_2_S responsible for the excellent properties. Interaction of H_2_S with the oxide surface is determined by two factors. First, hydrogen sulfide is a strong reducing agent: the value of the ionization potential of the H_2_S molecule is 4.10 eV. Secondly, heterolytic break of the S-H bond is quite easy, especially in the formation of new donor–acceptor bonds^30^. The important augmentation of the sensor signal is possible, if the interaction of high-resistance oxide with hydrogen sulfide results in reversible formation of a highly conducting sulfide^31^,^32^. In the case of Cu_2_O/Co_3_O_4_ nano/microstructure heteroarrays significant resistance change in the presence of H_2_S should be attributed to the formation of metallic Cu_2_S (Eq. 1). The products after sensing test were characterized by XPS for further confirming the formation of Cu_2_S, as the Fig. S2 shown. The sample were kept in 20 ppm H_2_S atmosphere in sealed box until XPS test. Fig. S2 (a) and (b) shows the high resolution Cu 2p and S 2p spectra of the Cu_x_S sulfurized after the sensing test, respectively. Fig. S2a presents two peaks at 932.4 and 952.2 eV corresponding to Cu 2p3/2 and Cu 2p1/2, respectively. The binding energies of the S 2p3/2 and S 2p1/2 peaks are 161.4 and 162.5 eV, respectively (Fig. S2b). These binding energies (BEs) are consistent with those previously reported for Cu and S in Cu_2_S^33^,^34^, confirming the chemical composition of Cu_2_S.


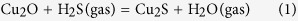



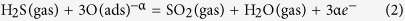


The Cu_2_O/Co_3_O_4_ nano/microstructure heteroarray is a multi-barrier system (as shown in Fig. 3c), where carriers transport through tunneling. However, the heterointerface barrier is sensitive to the carrier concentration, thus the tunneling modulation requires only a low level change of carrier concentration^35^. The tunneling modulation process is very rapid, even at and below room temperature^27^. When the sensor is exposed to H_2_S, part of the absorbed oxygen on the surface of Cu_2_O and Co_3_O_4_ are removed as the initial reaction mechanism, leaving oxygen-bound electrons in the surface (Eq. 2). As a result, the hole concentrations of p-type Cu_2_O and p-type Co_3_O_4_ decrease leading to a reduction of the carrier diffusion between Cu_2_O and Co_3_O_4_, which means an weaken of interface barrier. Finally, carriers have a relatively large probability of crossing the interface barrier. Subsequently, the remaining H_2_S continues to attach on to the surface of Cu_2_O, which give rise to the generation of Cu_2_S. At the critical H_2_S concentration (about 10 ppm in this case, as shown in Fig. 7c), the transformation of highly resistive Cu_2_O into metallic Cu_2_S can result in formation of conductance channels on the surface of the nanoarrays that leads to a drastic increase of the conductance. Therefore, at 10 ppm H_2_S atmosphere the signal of the sensor increases rapidly (a 10^2^-fold increase of current), and continues to increase as more parallel conductance channels are generated. This rapid increase in conductivity with increasing H_2_S pressure will then taper off as each conductance channel that is created becomes less important, resulting in a sigmoidal response curve presented in Fig. 4b. The two-stage response of sensor to H_2_S (Fig. 8) corresponding to the first stage of surface oxygen desorption and the second stage of Cu_2_S generation. When the sensor exposed in high H_2_S atmosphere, the number and the thickness of Cu_2_S conduct channel obvious increased due to the ample H_2_S supply. However the kinetics of converting copper sulfide to copper oxide is generally slow, so the recovering time will obviously increase.

The modulation of heterojunction barrier achieved in Cu_2_O/Co_3_O_4_ nano/microstructure heteroarrays is maximized. Compared with the always present interface barrier in previous reported CuO/Cu_2_O based heterostructure, the barrier can hide itself temporarily in Cu_2_O/Co_3_O_4_ nano/microstructure heteroarray when it exposed to H_2_S. The heterojunction barrier can be considered as a switch in galvanic circle of sensor (in air, the barrier is high, the carriers transport is difficult, the switch is off; in gas, the barrier is disappeared, the carriers transport is unobstructed, the switch is on). It is important to mention that the Cu_2_S conductance channel is throughout the nanoarrays without inserted heterojunction barriers. This advantage arises from the continuous distribution of Cu_2_O throughout the whole heteroarrays. This is the reason why our sensors have higher conductivity than others in the same conditions. Considering the above-mentioned factors, both the hidden heterojunction barrier and the Cu_2_S conductance channel responsible for the enhancement sensitivity at ultra-low temperature.

In summary, we have investigated both structure and H_2_S sensitivity of the Cu_2_O/Co_3_O_4_ nano/microstructure heteroarrays prepared by 2D electrodeposition technique. The detailed study of heteroarrays verifies that they are multi-barrier system with uniform cycle length and long-range order in millimeter scale. A parametric study was designed in order to understand the impact of the H_2_S concentration and temperature on the response signal. We found that the heteroarrays exhibit high sensitivity to H_2_S at and below room temperature, even as low as −30 °C. The detection limit can reach as low as sub-ppm at room temperature. The data confirm that both desorption of oxygen and generation of metallic Cu_2_S are responsible for the excellent sensitivity, and the generation of Cu_2_S conductance channel makes an important contribution to the signal enhance between 10 ppm and 80 ppm. The study provides a possibility to the facile prepared of gas sensor with high sensitivity at and below room temperature and a new direction of designing materials for ultra-low temperature gas sensing.

## Methods

Copper nitrate hydrate (Cu(NO_3_)_2_·3H_2_O, 99.9%), cobalt nitrate hexahydrate (Co(NO_3_)_2_·6H_2_O, 99.99%), nitric acid (HNO_3_, analytical-regent grade) were purchased from Aladdin. The solution was prepared with deionized water. All chemicals were used in the experiments without further purication.

The Cu_2_O/Co_3_O_4_ heterostructures were synthesized by an electrochemical deposition system in an ultra-thin electrolyte liquid layer. In a typical procedure, 50 mL of nitric acid solution (pH = 4.0) was first prepared, then 0.7248 g Cu(NO_3_)_2_ and 0.4365 g Co(NO_3_)_2_ were added into nitric acid solution. The silicon used in the experiment requires pre-processing, its surface was cleaned and oxidized to form an insulating SiO_2_ layer. First, the pre-treated silicon substrate (20 × 20 mm^2^) was placed onto a Peltier element on the bottom of growth chamber, and two parallel copper electrodes (30 μm thick, 99.99%) were put on the substrate separated by a distance of 6 mm. Then 20 μL prepared solution was dropped on the substrate between two electrodes. Afterwards, a cover glass was put on two electrodes carefully to make the space between cover glass and silicon substrate filled of electrolyte. By freezing the electrolyte at −4.5 °C, an ultra-thin ice layer (about 30 μm thick) could be formed between the silicon substrate and cover glass. Due to the partitioning effect, two ultra-thin liquid layer of concentrated electrolyte were formed between the ice and the silicon substrate as well as the ice and the cover glass separately. The thickness of the electrolyte layer is about 300 nm. Eventually, the deposition process was carried out by applying a 0.8 Hz semi-sine wave with amplitude varied from 0.5 to 1.5 V across the electrodes.

The morphology, nanostructure and interface electric field of Cu_2_O/Co_3_O_4_ nano/microstructure heteroarrays were characterized using field emission scanning electron microscopy (SEM, JEOL JSM-6700F), transmission electron microscopy (TEM, JEM-2010F) and scanning probe microscope (SPM, MicroNano D5A). The surface elemental composition was checked with X-ray photoelectron spectroscopy (XPS, ESCALAB MKII, VG).

The as-synthesized Cu_2_O/Co_3_O_4_ nano/microstructure heteroarrays were used as the sensing materials. The process of fabricating the sensor was described in detail in our previous work^10^. The gas-sensing properties to various concentrations of H_2_S were mearsured using a custom-made gas sensing system. The gas sensitivity is defined as the slope between gas response and gas concentration, and the response =(I_g_/I_a_) × 100, where I_a_ is the initial current in the air, and I_g_ is the current measured in the presence of H_2_S. The response time was defined as the time to reach 90% change in current after injecting H_2_S, and the recovery time was defined as the time needed for the sensor to return to 90% change in current upon removing H_2_S.

## Additional Information

**How to cite this article:** Cui, G. *et al*. Highly sensitive H_2_S sensors based on Cu_2_O/Co_3_O_4_ nano/microstructure heteroarrays at and below room temperature. *Sci. Rep.* **7**, 43887; doi: 10.1038/srep43887 (2017).

**Publisher's note:** Springer Nature remains neutral with regard to jurisdictional claims in published maps and institutional affiliations.

## Supplementary Material

Supplementary Information
