# Supplementary material for: Highly sensitive H2S sensors based on Cu2O/Co3O4 nano/microstructure heteroarrays at and below room temperature
Source: Sci Rep. 2017 Mar 2;7:43887. doi: 10.1038/srep43887 (PMC5333136; doi:10.1038/srep43887)
Supplement: Supplementary Information [file srep43887-s1.docx]

**Supplementary Information**

**Highly sensitive H_2_S sensors based on** **Cu_2_O/Co_3_O_4_ nano/microstructure heteroarrays at and below room temperature**

Guangliang Cui^1^, Pinhua Zhang^1^, Li Chen^1^, Xiaoli Wang^1^, Jianfu Li^2^, Changmin shi^1^ and Dongchao Wang^1^

**^1^** Institute of Condensed Matter Physics, Linyi University, Linyi, Shandong 276000, China.

**^2^** School of science, Linyi University, Linyi, Shandong 276000, China.

*Corresponding author: P. H. Zhang ([zhangpinhua@lyu.edu.cn](mailto:zhangpinhua@lyu.edu.cn))


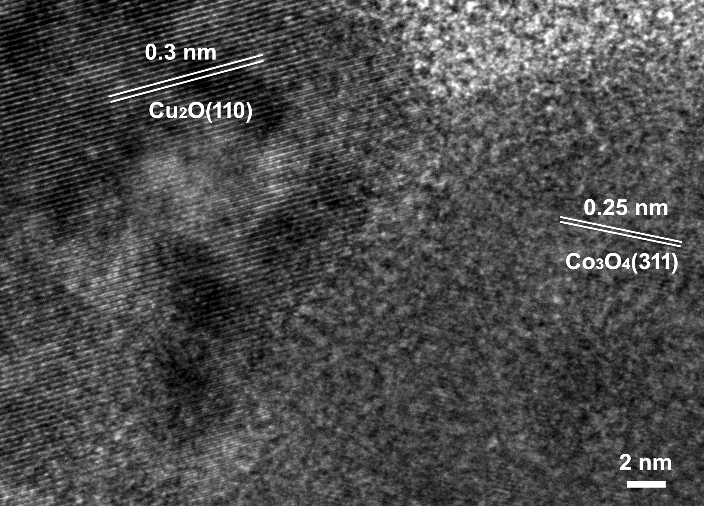


**Figure S1** HRTEM images of the Cu_2_O/Co_3_O_4_ nano/microstructure heteroarrays. The spacings of the fringes were measured to be 0.25 nm for Co_3_O_4_ and 0.3 nm for Cu_2_O.

The high resolution transmission electron microscopy (HRTEM) images of Cu_2_O/Co_3_O_4_ nano/microstructure heteroarrays was characterized and supplied in Fig. S1. The spacings of the fringes were measured to be 0.25 nm for Co_3_O_4_ and 0.3 nm for Cu_2_O, corresponding to the (311) plane of Co_3_O_4_ and the (110) plane of Cu_2_O. The results is agree with the SAED very well. Furthermore, Co_3_O_4_ and Cu_2_O are tight contact with each other, and the boundary is distinct.


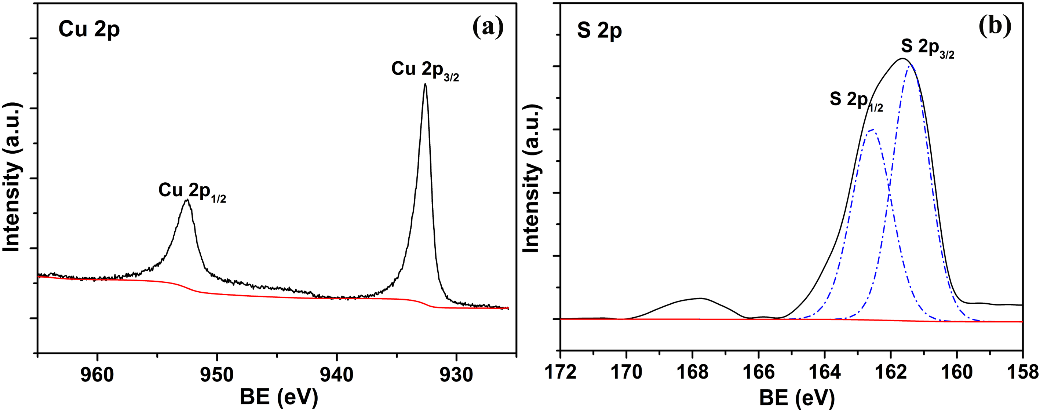


**Figure S2** High resolution Cu 2p (a) and S 2p (b) spectra of the Cu_2_O/Co_3_O_4_ nano/microstructure heteroarrays after the sensing test.

The XPS was performed to confirm the formation of Cu_2_S and examine the accurate chemical composition of the sulfurized Cu. Fig. S2 (a) and (b) shows the high resolution Cu 2p and S 2p spectra, respectively, of the Cu_x_S sulfurized after the sensing test. Fig. S2a presents two peaks at 932.4 and 952.2 eV corresponding to Cu 2p_3/2_ and Cu 2p_1/2_, respectively. The binding energies of the S 2p_3/2_ and S 2p_1/2_ peaks were 161.4 and 162.5 eV, respectively (Fig. S2b). These binding energies (BEs) are consistent with those previously reported for Cu and S in Cu_2_S^R1^, confirming the chemical composition of Cu_2_S.

*
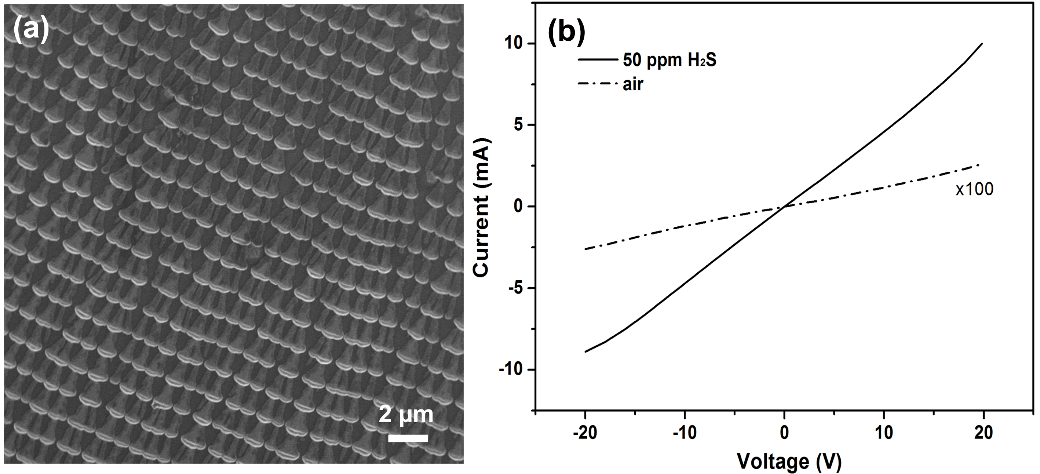
*

**Fig. S3** The response of sensor based on pure Cu_2_O to 50 ppm H_2_S in air at working temperature of 15 ^o^C, and the current in air was magnified 100 times.

Pure Cu_2_O nano/microstructure heteroarrays were prepared under the same experimental conditions. The SEM picture (Fig. S3a ) of pure Cu_2_O nano/microstructure heteroarrays exhibits a similar morphology with Cu_2_O/Co_3_O_4_ nano/microstructure heteroarrays. The *I-V* curves of sensor based on pure Cu_2_O to 50 ppm H_2_S in air at working temperature of 15 ^o^C was shown in Fig. S3b. The response of this sensor is 380 when the bias is 20 V, which is much less than the Cu_2_O/Co_3_O_4_ nano/microstructure heteroarrays’ (Fig. 4b).
